# Effects of elevated carbon dioxide on plant growth and leaf photosynthesis of annual ryegrass along a phosphorus deficiency gradient

**DOI:** 10.3389/fpls.2023.1271262

**Published:** 2023-11-27

**Authors:** Fei Li, Chunlin He, Zhijie Chang, Chao Ma, Jingjin Yu, Liang Liu, Yunxin Zhang, Lihua Hao

**Affiliations:** ^1^ School of Water Conservancy and Hydropower, Hebei University of Engineering, Handan, China; ^2^ Jiangsu Provincial Flood Control and Drought Relief Center, Nanjing, China; ^3^ College of Water Resources and Architectural Engineering, Northwest A&F University, Yangling, China; ^4^ School of Agro-Grassland Science, Nanjing Agricultural University, Nanjing, China

**Keywords:** elevated CO_2_ concentration, P limitation, stomatal traits, leaf photosynthesis, biochemical

## Abstract

**Introduction:**

Soil phosphorus (P) deficiency limits plant growth and productivity in grassland ecosystems and may moderate the growth-promoting effects of “carbon dioxide (CO_2_) fertilization effect”.

**Methods:**

To evaluate the interactive effects of these two factors on the growth and physiology for annual ryegrass (*Lolium multiflorum* Lam.), plants were grown in controlled growth chambers with a range of P supply (0.004, 0.012, 0.02, 0.06, 0.1 and 0.5 mM) under two levels of CO_2_ (400 and 800 μmol mol^-1^, respectively).

**Results:**

Elevated [CO_2_] dramatically increased the aboveground biomass and net photosynthetic rates of annual ryegrass by 14.5% and 25.3% under sufficient P supply (0.5 mM), respectively, whereas decreased the belowground biomass and net photosynthetic rates under lower P supply of P_0.004_, P_0.02_, and P_0.06_. Two-way ANOVA results showed that CO_2_ × P (*p* < 0.001) significantly affected stomatal traits, leaf photosynthesis and biomass. The stimulation of growth and photosynthesis by elevated CO_2_ concentration (*e*[CO_2_]) was reduced or highly suppressed, indicating that the sensitivity of annual ryegrass to P deficiency was enhanced under *e*[CO_2_].

**Discussion:**

These results indicated that P limitation may offset the positive effects of *e*[CO_2_] on plant growth by altering stomatal traits, leaf photochemical processes and biochemical composition in annual ryegrass.

## Introduction

Global atmospheric carbon dioxide concentration ([CO_2_]) has dramatically been accelerated with an average growth rate of about 1.6 μmol mol^-1^ from 280 μmol mol^-1^ to 400 μmol mol^-1^ in recent past five decades (IPCC, 2013). Meanwhile, many climate models have also predicted that the atmospheric [CO_2_] would go up to 800 μmol mol^-1^ by the end of this century (IPCC, 2013). It has been well demonstrated that *e*[CO_2_] stimulated plant growth (Suter et al., 2002; Ainsworth, 2008; Wang and Taub, 2010; Yu et al., 2012b) through the “CO_2_ fertilization effect” by affecting physiological and biochemical processes (Taub and Wang, 2008; Yu et al., 2012a; Arndal et al., 2014) such as photosynthesis (Leakey et al., 2006; Leakey et al., 2009; Zhang et al., 2010; Zheng et al., 2018) and respiration (Crous et al., 2011; Tan et al., 2013), especially for the C_3_ plants (Lee et al., 2001; Ainsworth and Rogers, 2007; Zheng et al., 2019). Nevertheless, plants in response to *e*[CO_2_] varied with nutrient availability, and the CO_2_ fertilization effect generally declined in parallel with the decreases of nutrient availability (Menge and Field, 2007; McCarthy et al., 2010; Norby et al., 2010; Lenka and Lal, 2012; Pandey et al., 2015; Zhang et al., 2017). Thereby, the CO_2_ fertilization effect on plant growth might be mitigated or even counteracted by the limitation of nutrient availability due to the higher nutrient demand of plants with rising atmospheric CO_2_ (Lenka and Lal, 2012; Pandey et al., 2014; Jin et al., 2015; Ellsworth et al., 2017). For instance, Lewis et al. (2010) analyzed the data from *Populus deltoides* and pointed out that increasing CO_2_ nearly doubled the total biomass under 0.5 mM P supply, while it increased by only 7% under the heaviest P deficiency (0.004 mM). Overall, elevated [CO_2_] and nutrient availability may have confounding impacts on plant growth and biomass allocation, and thus investigating the potential processes by which nutrient supply regulate the CO_2_ fertilization effect on plant growth is critical to predicting the impacts of future climate change on the net primary productivity (NPP) of terrestrial ecosystems, particularly in the natural ecosystems such as forests and grasslands, which are limited by nutrient availability (Kimball et al., 2002; Sakurai et al., 2014; Deng et al., 2017).

Phosphorus (P) is an extremely critical nutrient for sustaining plant growth, development and reproduction (Chiera et al., 2002; Nord and Lynch, 2009; Peñuelas et al., 2013; Jin et al., 2015; Zhan et al., 2017). Because P plays a vital role not only in diverse biochemical processes, such as cell and lipid metabolism (Vance et al., 2003), but also serves as an essential source of energy for numerous biological functions (Almeida et al.,1999; Abel et al., 2002; Lambers et al., 2006). However, soil P deficiency is common in terrestrial ecosystems and is also most likely to become worse under future climate change, where rising [CO_2_] may increase the required amount of P for sustaining plant growth (Elser et al., 2007; Richardson et al., 2009). Meanwhile, soil P availability is becoming lower as global reserves deplete (Fay et al., 2015; Jin et al., 2015). The diminishing P availability may gradually become a major limiting nutrient on plant growth in managed and natural ecosystems under elevated [CO_2_] (Vance et al., 2003; Lewis et al., 2010; Lenka and Lal, 2012; Singh et al., 2013a). While most of previous studies investigating the effects of nutrient supply on plant responses to elevated [CO_2_] have focused primarily on nitrogen (N) limitation for leaf photosynthesis (Hungate et al., 2003; Lewis et al., 2004; Ainsworth and Long, 2005; Reich et al., 2006; Xu et al., 2013), P availability in response to elevated [CO_2_] is likely to be particularly important (Jin et al., 2015).

It is well demonstrated that P supply regulates the plant response to *e*[CO_2_] and is intrinsically triggered by leaf photosynthesis (Duchein et al., 1993; Norisada et al., 2006; Singh et al., 2013b; Sinhg and Reddy, 2014), which is highly related to the changes in stomatal diffusion processes as well as the biochemical and photochemical processes under higher [CO_2_] (Jacob and Lawlor, 1991; Singh et al., 2013b). Previous research have established that the responses of leaf photosynthesis to *e*[CO_2_] may be affected by low P availability through decreasing stomatal conductance (Kirschbaum and Tompkins, 1990; Singh et al., 2013a). Moreover, low P limitation may also affect the biochemical and photochemical processes of leaf photosynthesis in response to elevated [CO_2_] by the regeneration of triose-phosphate utilization (TPU) during ribulose bisphosphate (RuBP) regeneration (Rogers et al., 1993; Wissuwa et al., 2005; Pandey et al., 2015). Meanwhile, soil P deficiency may lower the activity of Calvin cycle enzymes, thus directly limit photosynthetic capacity under rising [CO_2_] (Palma et al., 2000). Additionally, the photosynthetic responses to elevated [CO_2_] can also be affected by low soil P supply through limiting plant growth and biomass allocation between source and sink tissues (Fredeen et al., 1989; Pandey et al., 2015). Understanding the potential mechanisms that low P availability affects photosynthetic responses to rising [CO_2_] is critical for assessing the impacts of elevated [CO_2_] on the structure and function of terrestrial ecosystems limited by low P supply under future climate change scenarios.

Grasslands hold a significant position within terrestrial ecosystems, as their responses to elevated [CO_2_] play a pivotal role in the global carbon-water cycling (Coleman et al., 1993; Steffen and Canadell, 2005). The plant coverage and net primary production of grasslands are usually limited by soil P availability under elevated [CO_2_] (Elser et al., 2007; Fay et al., 2015; Ceulemans et al., 2017). Annual ryegrass (*Lolium multiflorum* Lam.) is one of the most important principal forages with considerable ecological and economic significances due to high yield and quality in temperate grasslands and pastures (Li et al., 2007; Wang et al., 2013; Castanheira et al., 2014). In these contexts, low soil P supply may be a major factor limiting the CO_2_ fertilization effect on plant growth and leaf photosynthesis of annual ryegrass under future climate change (Byrne et al., 2011; Xu, 2015; Zheng et al., 2018). Nevertheless, most of previous studies regarding the plant responses to elevated [CO_2_] and P supply are primarily focused on trees (Lewis et al., 2010; Duan et al., 2019) and crops (Wissuwa et al., 2005). Thus, it is necessary to quantify whether P supply will affect grass growth and photosynthesis through altering the physiological and biochemical processes under enriched [CO_2_]. Consequently, it is unclear whether grasses response to rising [CO_2_] vary with P supply, even few studies have examined the responses of plant growth and leaf photosynthesis to elevated [CO_2_] in grass species with P deficiency (Edwards et al., 2006). Understanding the underlying mechanisms and processes of low soil P availability on plant growth and biomass allocation of annual ryegrass with changes in stomatal traits, leaf photosynthesis and plant biochemistry under elevated [CO_2_] may have important significance on projecting the net primary productivity (NPP) and guiding the formulation of adaptation policies for grasslands.

The aims of this study are to: (1) examine the combined effects of *e*[CO_2_] and soil P deficiency on the annual ryegrass growth and biomass allocation.; (2) investigate the potential processes that low P availability affecting photosynthetic responses to elevated [CO_2_] in annual ryegrass; (3) explore the underling mechanisms that soil P deficiency regulating CO_2_ fertilization effect on annual ryegrass growth with changes in stomatal traits, leaf photosynthesis and biochemistry.

## Materials and methods

### Growth chamber experiments

A golf hole cutter was utilized to eliminate the effect of errors in initial aboveground and belowground biomass (10 cm diameter × 20 cm long). Annual ryegrass was transplanted in the experimental farm at Hebei University of Engineering, Handan City, Hebei Province, China. Then, the collected grasses were transplanted into pots (10 cm diameter × 100 cm long) filled with fritted clay and moved to artificial climate chambers (Model BDP-2000, Ningbo Prandt Instrument Co., Ltd, China). We trimmed grasses every 30 days to a 5-cm canopy height during the 90 days experimental treatments to keep grass plants in good growth condition (Yu et al., 2012b).

Eight artificial climate chambers were utilized to automatically monitor and control CO_2_, four of which were set as modern CO_2_ (*a*[CO_2_]; 400 μmol mol^-1^) and the remaining four were set as elevated CO_2_ (*e*[CO_2_]; 800 μmol mol^-1^). The environmental settings for all eight environmental growth chambers were at 25/20°Cday/night temperature, 800 μmol m^-2^ s^-1^ PAR canopy light intensity, 65% relative humidity, and a 12-h photoperiod of 7:00-19:00. To minimize confounding effects of environmental variation between two chambers, we changed the [CO_2_] of each growth chamber every 7 days, and then relocated the CO_2_ treated annual ryegrass plants to the growth chambers with corresponding [CO_2_] during the whole experiment. In each artificial climate chamber, six randomly selected pots of annual ryegrass plants were watered to through-flow twice a week with half-strength Hoagland’s solution modified to generate six P concentration treatments of 0.004, 0.012, 0.02, 0.06, 0.1-, and 0.5-mM P as KH_2_PO_4_, respectively. To ensure that all grasses have the same amount of potassium kalium (K) in the nutrient solution at each watering, we add an additional moderate amount of KCL to supplement the K in the half-strength Hoagland’s solution. Four artificial climate chambers with *a*[CO_2_] or *e*[CO_2_] are biological replications (n = 4).

### Measuring stomatal density, morphological traits and distribution pattern of stomata

To characterize the maximum stomatal pore size of annual ryegrass, we selected recently expanded leaves for sampling stomatal imprints from the middle section on the abaxial surface using colorless nail varnish in artificial climate chambers on the 30th, 60th, and 90th days after CO_2_ treatment and P treatments (Zheng et al., 2013; Xu, 2015). We observed and photographed the collected imprints using the method of Zheng et al. (2013) and measured the stomatal aperture length (SAL), stomatal aperture width (SAW), stomatal aperture circumference (SAC) and stomatal aperture area (SAA) using the Image J quantification software (NH, Bethesda, MD). Stomata on each surface were counted and combined for calculating stomatal density (SD) (Ceulemans et al., 1995) and the stomatal aperture shape index (SASI) was also calculated as $\sqrt{\text{SAA}}\text{/SAC}\times 100\%$ . The morphological traits of stomata were visualized and photographed with a scanning electron microscopy (FEI Corp, USA). We randomly selected four images (a magnification of 100) from each treatment to estimate the stomatal spatial distribution pattern. The selected images were digitized with a GIS software (ArcGIS 10.0; ESRI Inc., Redlands, CA). In this study, the center of each stoma was treated as a single point. Then the point pattern analysis was conducted with the Ripley’s *K*-function (Ripley, 1976). Comprehensive guidelines for the analysis of stomatal spatial distribution pattern can be found in Xu (2015) and Zheng et al. (2020).

### Measuring leaf gas exchange

A portable LI-6400 photosynthesis system (Li-Cor Inc., Lincoln, NE, USA) was utilized to determine the net photosynthetic rate (*P*_n_), stomatal conductance (*G*_s_) and transpiration rate (*T*_r_) on recently expanded leaves on the 30th, 60th, 90th days after CO_2_ treatment and P treatments. All measurements were performed in the standard cuvette chamber (2 cm × 3 cm) with the CO_2_ concentration of 400 μmol mol^-1^, the saturating light at 1000 μmol photons m^-2^ s^-1^, the leaf-to-air vapor pressure deficit (VPD) of 1.5 KPa and the temperature of 20°C. The intrinsic water use efficiency (*WUE*) was calculated as *P*_n_/*T*_r_.

### Measuring plant biomass and analyzing tissue carbon, nitrogen and phosphorus contents

The aboveground and belowground biomass were harvested using the physical cutting at the end of the 90-day experiment and oven-dried the separated tissues at 80°C to a constant weight. Finally, the data of biomass were weighed using an electronic scale. The aboveground and belowground portions were grinded to fine powder using a ball mill (MM2, Fa. Retsch, Haan, Germany). The tissue phosphorus (P), carbon (C), and nitrogen (N) contents of shoots and roots were determined using an elemental analyzer (Vario Max CN; Elemnetar Corp., Germany). All the biochemical analyses were repeated four times (n = 4).

### Statistical analysis

Two-way analysis of variance (ANOVA) was utilized to test the interactive effects of P concentration and [CO_2_] on plant biomass, stomatal traits, and leaf gas exchange as well as the contents of P, C, and N among different treatments (*p* < 0.05). Additionally, a three-way analysis of variance (ANOVA) was also used to estimate the interactive effects of [CO_2_] × P × plant tissues on P, C, N contents (*p* < 0.05). Furthermore, we used linear and non-linear regressions to analyze the relationship between biomass and other variables (*p* < 0.05). All statistical analyses were conducted using the SPSS 20.0 software (Chicago, IL, USA).

## Results

### Effects of P supply and [CO_2_] on the plant biomass of annual ryegrass

**Figure 1:**
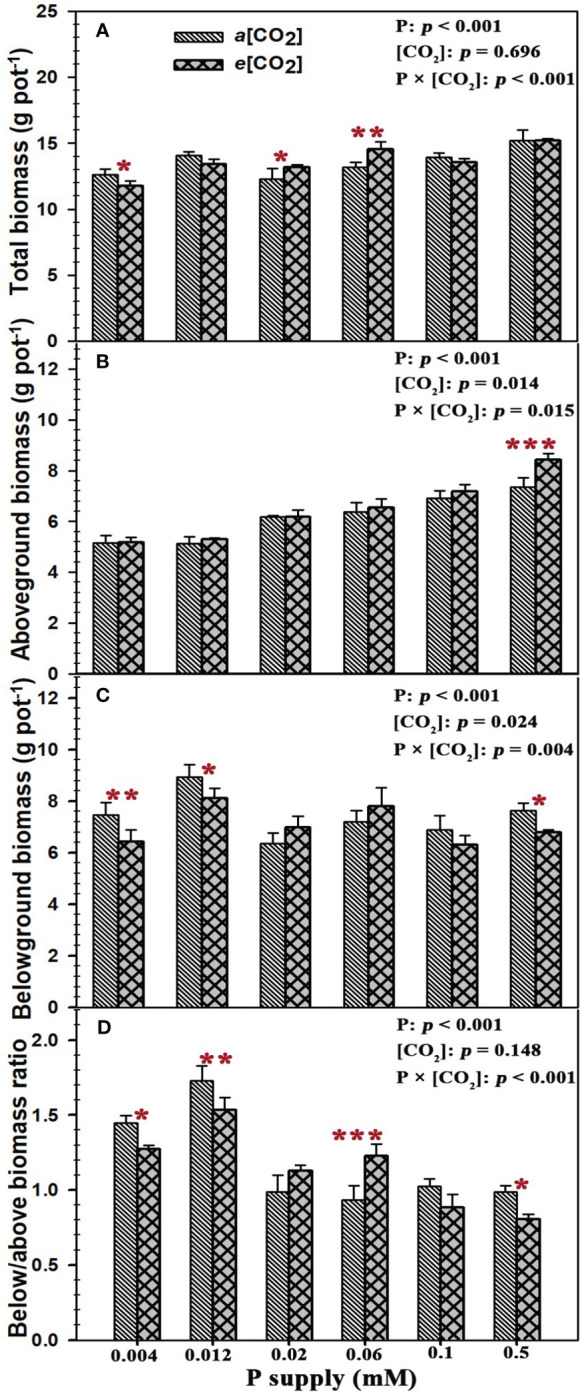
Effects of elevated [CO_2_] on plant biomass and its allocation of annual ryegrass under P deficits. Note that the grey bars represent ambient CO_2_ concentration (Ca) and the black bars represent elevated CO_2_ concentration (Ce). The symbols *, **, and *** indicate that the significant differences between Ca and Ce under the same P treatment. The part labels are mean that ANOVA p-values for P and [CO_2_] and interactive effects of [CO_2_] and P on annual ryegrass biomass.

Our results showed that the total plant biomass, aboveground biomass, belowground biomass and below/above biomass ratio of annual ryegrass were substantially changed by P supply (all *p* < 0.001), while *e*[CO_2_] only remarkably affected the aboveground biomass (*p* = 0.014) and belowground biomass (*p* = 0.024; **Figure 1**). Specifically, *e*[CO_2_] marginally decreased the total plant biomass by 6.6% at P_0.004_ (*p* < 0.05), but the total plant biomass under the P concentrations of 0.02 Mm (P_0.02_) and 0.06 mM (P_0.06_) was obviously increased by 7.1% (*p* < 0.05) and 10.4% (*p* < 0.01) (**Figure 1A**). Moreover, elevated [CO_2_] dramatically increased the aboveground biomass by 14.5% (*p* < 0.001) under the highest P concentration of 0.5 mM (P_0.5_) (**Figure 1B**). By contrast, rising [CO_2_] decreased the belowground biomass by 13.7% (*p* < 0.01), 9.2% (*p* < 0.05), and 11.1% (*p* < 0.05) under the P concentrations of 0.004 mM (P_0.004_), 0.012 mM (P_0.012_), and 0.5 mM (P_0.5_) (**Figure 1C**). Similar to the changes in belowground biomass, elevated [CO_2_] also changed the allocation of plant biomass between belowground and aboveground (below/above biomass ratio) with decreasing the below/above biomass ratio by 11.9%, 11.4%, and 18.5% at P_0.004_ (*p* < 0.05), P_0.012_ (*p* < 0.01), and P_0.5_ (*p* < 0.05), while the below/above biomass ratio of annual ryegrass at P_0.006_ was substantially enhanced by 31.5% under elevated [CO_2_] (*p* < 0.001; **Figure 1D**). Moreover, our one-way ANOWA results revealed that the total biomass (*p* < 0.001), aboveground biomass (*p* < 0.001), belowground biomass (*p* < 0.001), and the ratio of below/above biomass (*p* < 0.001) were significantly changed by the P deficiency, while *e*[CO_2_] only marginally effected the aboveground biomass (*p* = 0.014) and belowground biomass (*p* = 0.024) of annual ryegrass (**Figure 1**). Additionally, the remarkably interactive effects of P and [CO_2_] were found on the total plant biomass, aboveground biomass, belowground biomass, and below/above biomass ratio of annual ryegrass (**Figure 1**).

### Effects of P supply and [CO_2_] on leaf gas exchange of annual ryegrass

**Figure 2:**
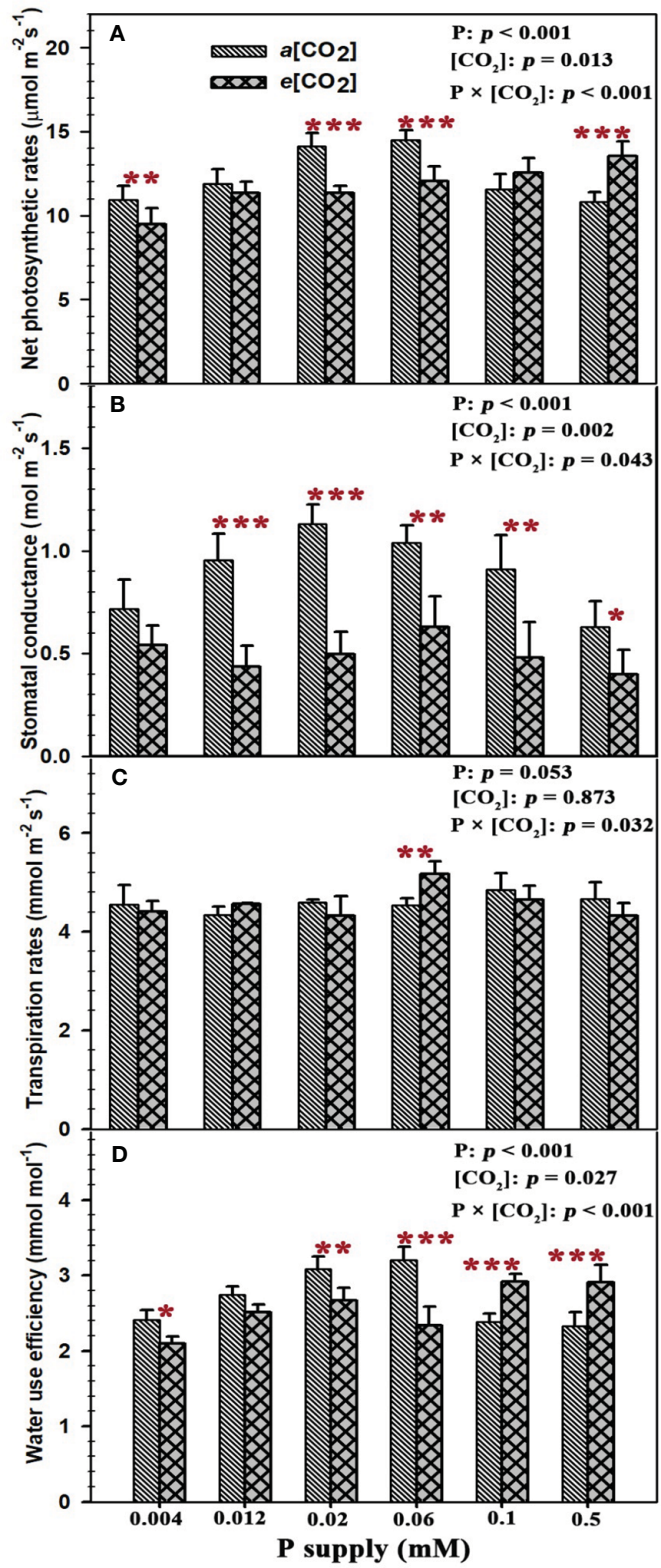
Effects of elevated [CO_2_] on leaf gas exchange of annual ryegrass under P deficits. Note that the grey bars represent ambient CO_2_ concentration (Ca) and the black bars represent elevated CO_2_ concentration (Ce). The symbols *, **, and *** indicate that the significant differences between Ca and Ce under the same P treatment. The part labels are mean that ANOVA p-values for P and [CO_2_] and interactive effects of [CO_2_] and P on leaf exchange of annual ryegrass.

Sufficient P supply (P_0.5_) had a strong CO_2_ fertilization effect on the net photosynthetic rates of annual ryegrass, as evidenced by the 25.3% increase of net photosynthetic rates (*p* < 0.001) (**Figure 2A**). By contrast, the net photosynthesis rates of annual ryegrass at lower P supply of P_0.004_, P_0.02_, and P_0.06_ were significantly decreased by 13.6% (*p* < 0.01), 19.8% (*p* < 0.001) and 16.9% (*p* < 0.001) under elevated [CO_2_]. Meanwhile, elevated [CO_2_] substantially reduced the stomatal conductance by 54.2% (*p* < 0.001), 56.0% (*p* < 0.001), 39.6% (*p* < 0.01), 47.3% (*p* < 0.01), and 36.7% (*p* < 0.05) at the P treatments of P_0.012_, P_0.02_, P_0.06_, P_0.1_, and P_0.5_ except for the stomatal conductance under the P supply of P_0.004_ (**Figure 2B**). However, elevated [CO_2_] only increased the leaf transpiration rate at P_0.06_ by 14.1% (*p* < 0.01), and barely affected the leaf transpiration rates under other P treatments (**Figure 2C**). Consequently, *e*[CO_2_] dramatically reduced the water use efficiency of annual ryegrass by 13.1% (*p* < 0.05), 13.5% (*p* < 0.01), and 27.1% (*p* < 0.001) under lower P treatments of P_0.004_, P_0.02_ and P_0.06_ (**Figure 2D**), whereas the water use efficiency at higher P treatments of P_0.1_ and P_0.5_ was significantly enhanced by 22.3% (*p* < 0.001) and 24.8% (*p* < 0.001; **Figure 2D**). Moreover, the significantly interactive effects of P supply and *e*[CO_2_] were also found on the net photosynthetic rates (*p* < 0.001), stomatal conductance (*p* = 0.043), transpiration rates (*p* = 0.032) and water use efficiency (*p* < 0.001) of annual ryegrass (**Figure 2**).

### Effects of P supply and [CO_2_] on the morphological traits of individual stoma and the spatial distribution pattern of stomata on annual ryegrass leaves

**Figure 3:**
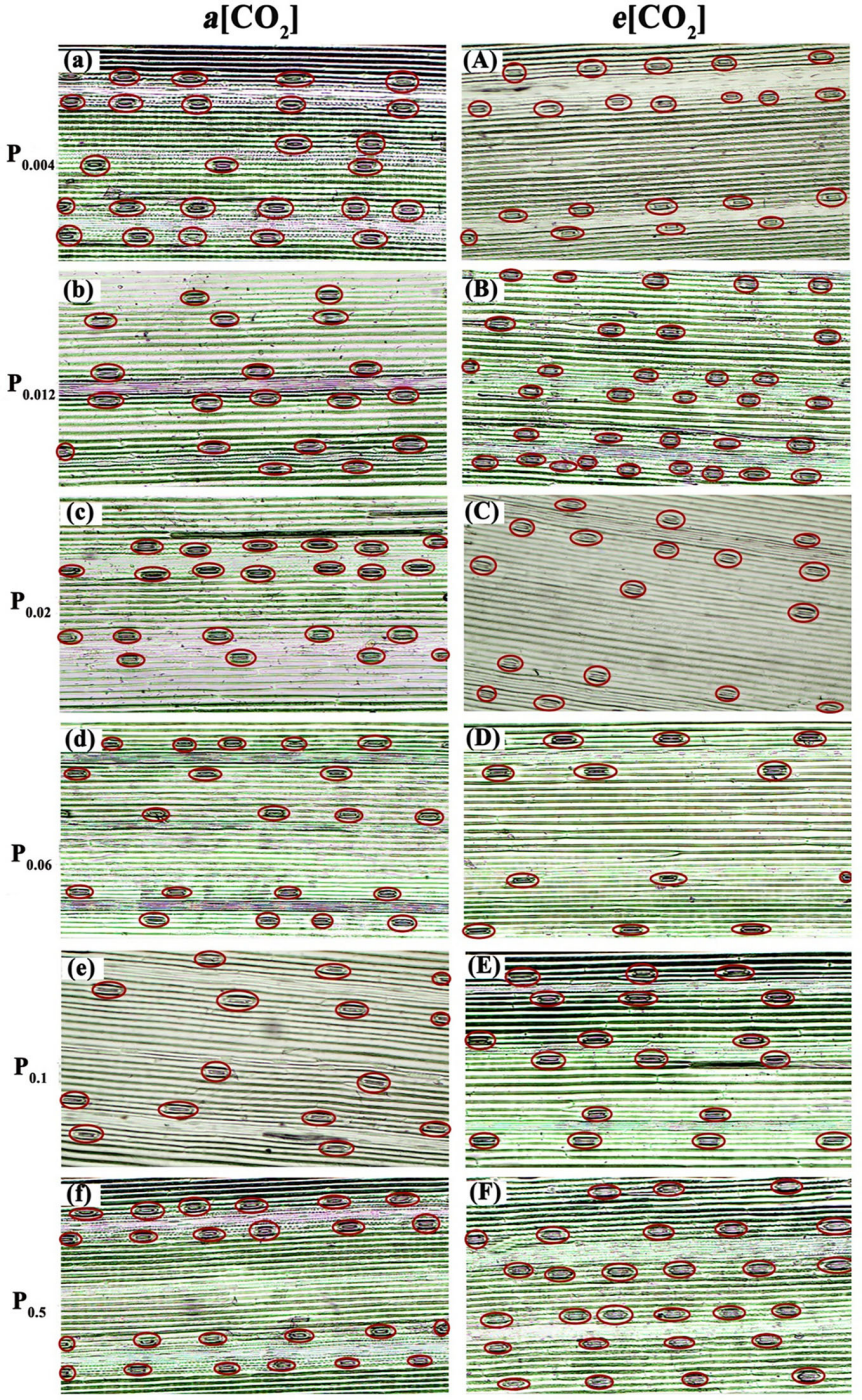
Observation on stomatal density of annual ryegrass under light microscopy. The P supply are 0.004 **(a)**, 0.012 **(b)**, 0.02 **(c)**, 0.06 **(d)**, 0.1 **(e)**, and 0.5 **(f)** mM under ambient CO_2_, respectively;the P supply are 0.004 **(A)**, 0.012 **(B)**, 0.02 **(C)**, 0.06 **(D)**, 0.1 **(E)**, and 0.5 **(F)** mM under elevated [CO_2_], respectively.

**Figure 4:**
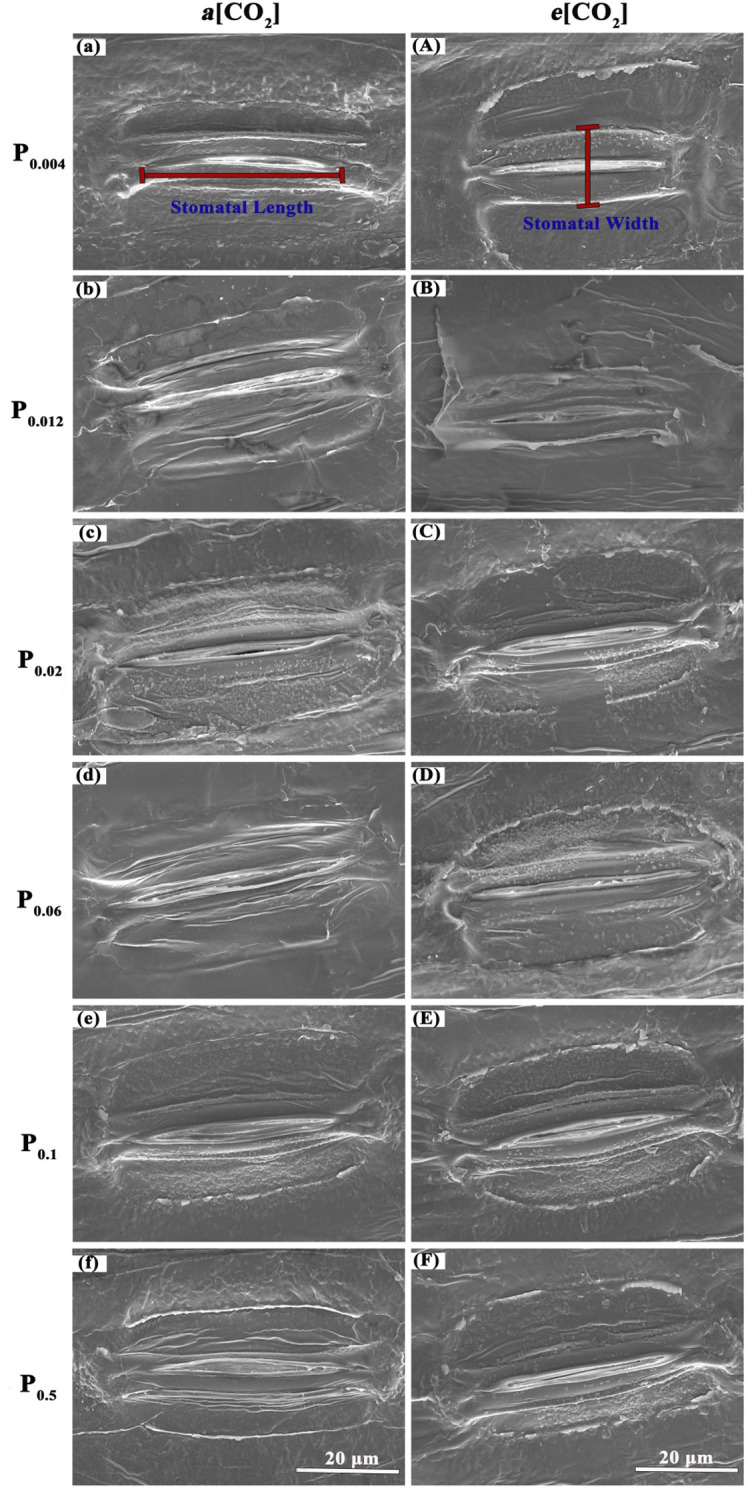
Micrographs of stomatal morphology photographed with Scanning Electrical Microscopy (SEM). The P supply are 0.004 **(a)**, 0.012 **(b)**, 0.02 **(c)**, 0.06 **(d)**, 0.1 **(e)**, and 0.5 **(f)** mM under ambient CO_2_, respectively;the P supply are 0.004 **(A)**, 0.012 **(B)**, 0.02 **(C)**, 0.06 **(D)**, 0.1 **(E)**, and 0.5 **(F)** mM under elevated [CO_2_], respectively.

Elevated [CO_2_] substantially affected the stomatal density (SD) of annual ryegrass regardless of P supply (**Table 1**). Specifically, *e*[CO_2_] dramatically increased the SD by 72.3%, 34.8% and 25.6% under P_0.012_, P_0.1_, and P_0.5_, whereas obviously decreased the SD by 17.1%, 27.1%, and 35.5%, respectively, at the P supply of P_0.004_, P_0.02_, and P_0.06_ (all *p* < 0.05; **Table 1**; **Figure 3**). Moreover, elevated [CO_2_] dramatically decreased the stomatal area (SA) by 13.7%, 12.5% and 11.5% at P_0.004_, P_0.1_, and P_0.5_ (all *p* < 0.05), which may be due to the smaller stomatal length and width (**Table 1**; **Figure 4**), and the minimum and maximum values of the stomatal area were occurred at P_0.012_ and P_0.1_, respectively (**Table 1**; **Figure 4**). Our two-way ANOVA results showed that the SD of annual ryegrass was substantial changed by [CO_2_] or P supply (**Table 2**). Additionally, [CO_2_] × P supply also significantly affected the SD, SAL, SAW, SAA, and SAA (all *p* < 0.05), but barely changed the SAC (**Table 2**).

**Table 1:** Effects of elevated [CO_2_] on the stomatal morphology of annual ryegrass under P deficits.

Stomatal morphology	*a*[CO_2_]						*e*[CO_2_]					
	P_0.004_	P_0.012_	P_0.02_	P_0.06_	P_0.1_	P_0.5_	P_0.004_	P_0.012_	P_0.02_	P_0.06_	P_0.1_	P_0.5_
**Stomatal density (No. mm^-2^)**	24.6 ± 1.5c	19.9 ± 1.7e	20.8 ± 1.4de	20.7 ± 2.3de	20.7 ± 2.3de	22.8 ± 2.4cd	20.4 ± 1.0de	34.2 ± 0.2a	15.2 ± 1.8f	13.3 ± 0.3f	19.7 ± 1.9e	28.6 ± 0.3b
**Stomatal length (μm)**	42.6 ± 2.4bcd	44.8 ± 1.7bc	44.4 ± 1.7bc	42.6 ± 2.9bcd	48.4 ± 4.3a	42.6 ± 2.8bcd	41.3 ± 2.1bcd	40.0 ± 2.9d	41.2 ± 1.0bcd	45.1 ± 0.4ab	43.6 ± 1.4bcd	40.9 ± 2.6cd
**Stomatal width (μm)**	4.2 ± 0.1cde	4.2 ± 0.3cde	4.2 ± 0.2cde	4.1 ± 0.3de	5.1 ± 0.4a	4.6 ± 0.1b	4.0 ± 0.2e	4.3 ± 0.1bcde	4.5 ± 0.4bc	3.9 ± 0.2e	4.5 ± 0.3bcd	4.3 ± 0.1bcde
**Stomatal perimeter (μm)**	87.2 ± 6.7b	91.7 ± 2.6b	94.3 ± 6.5b	90.1 ± 4.6b	105.8 ± 8.3a	93.4 ± 6.7b	87.1 ± 4.7b	86.3 ± 5.9b	85.6 ± 2.8b	90.0 ± 4.3b	94.7 ± 3.3b	86.4 ± 5.7b
**Stomatal area (μm^2^)**	182.3 ± 7.8b	151.3 ± 11.2de	154.2 ± 13.3d	156.5 ± 11.1d	201.1 ± 12.7a	182.3 ± 10.7b	157.3 ± 14.7d	136.7 ± 8.4e	158.1 ± 7.4d	166.6 ± 3.8bcd	175.9 ± 4.1bc	161.2 ± 9.8cd
**Stomatal shape index**	0.16 ± 0.01a	0.13 ± 0.006c	0.13 ± 0.004c	0.14 ± 0.006c	0.13 ± 0.01c	0.15 ± 0.011b	0.14 ± 0.008c	0.14 ± 0.005c	0.15 ± 0.006b	0.14 ± 0.007c	0.14 ± 0.005c	0.15 ± 0.008b

Different lowercase letters indicate significant differences between P deficits treatments at 0.05 level.

**Table 2:** ANOVA *p*-values for the effects of P and CO_2_ and interactive effects of P and [CO_2_] on the stomatal morphology of annual ryegrass.

Stomatal traits	Stomatal density	Stomatal length	Stomatal width	Stomatal perimeter	Stomatal area	Stomatal shape index
[CO_2_]	***p*<0.05**	***p*<0.05**	*p*=0.09	***p*<0.05**	***p*<0.001**	***p*** =0.19
P supply	***p*<0.001**	***p*<0.05**	***p*<0.001**	***p*<0.05**	***p*<0.001**	***p*<0.05**
[CO_2_] × P supply	***p*<0.001**	***p*<0.05**	***p*<0.05**	*p*=0.261	***p*<0.05**	***p*<0.05**

P<0.05 were considered significant and highlighted in bold.

**Figure 5:**
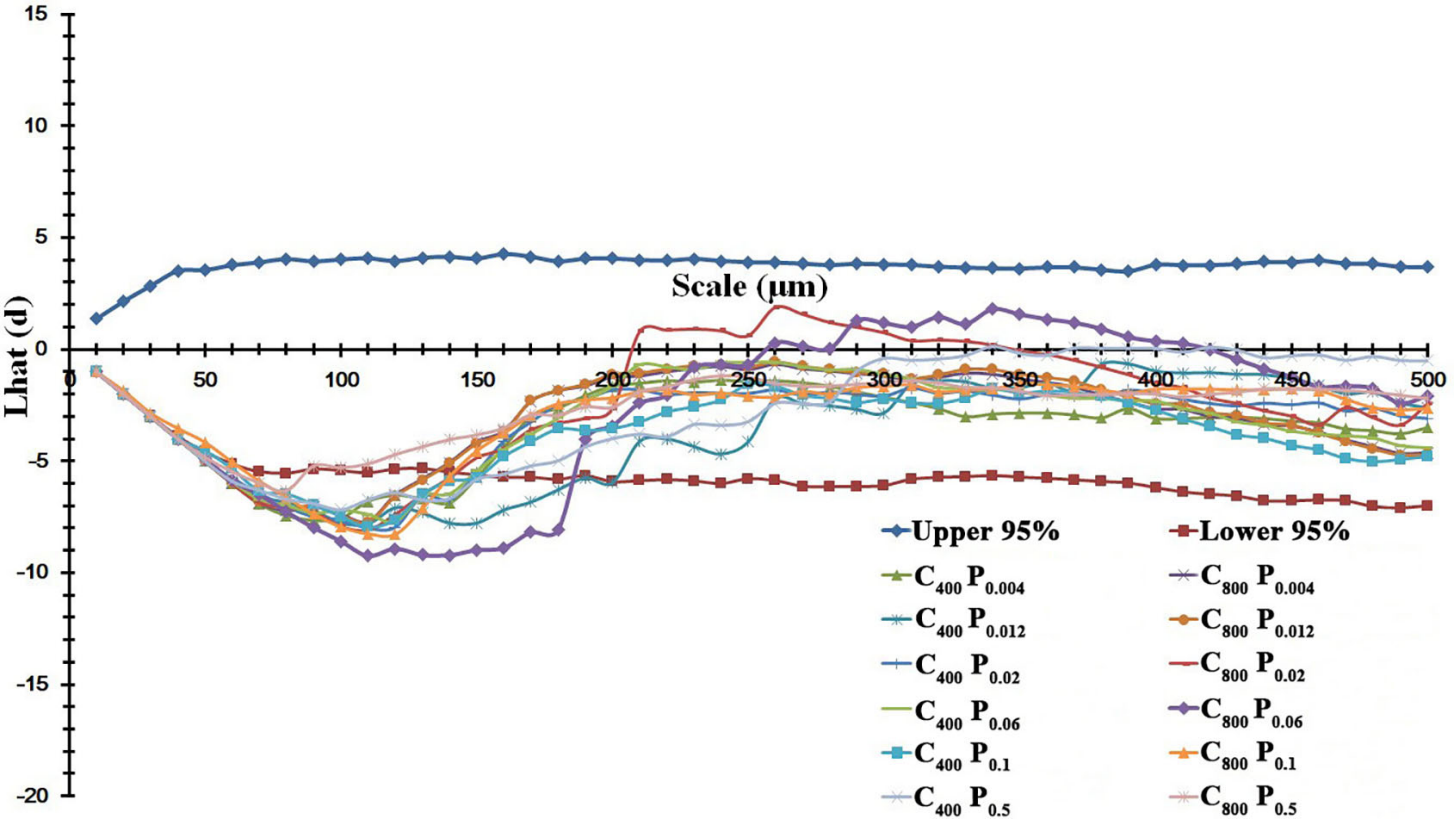
Effects of elevated [CO_2_] on the spatial distribution pattern of stomata under P deficits. Note: that the more regular distribution pattern of stomata featured with a lower Lhat(d) value. The upper and lower 95% boundaries were obtained by Monte Carlo simulation of 1000 replicates.

The spatial distribution pattern of annual ryegrass was also changed by P supply and *e*[CO_2_] (**Figure 5**). In general, the spatial pattern of stomata distributed on leaves of annual ryegrass followed a regular pattern at small scales (<150 µm) and a random distribution at larger scales (>200 µm) regardless of [CO_2_] and P supply (**Figure 5**). Interestingly, the most regular pattern both at the scale of *c.* 110 μm regardless of the [CO_2_] concentration was observed in the current study, as evidenced by the average minimum Lhat(d) values of -9.24 under *a*[CO_2_] and -8.00 under *e*[CO_2_] (**Figure 5**). Moreover, *e*[CO_2_] produced more regular spatial patterns of stomata at small scales when annual ryegrass was subjected to three higher P supply of P_0.06_, P_0.1_, and P_0.5_, due to the lower Lhat(d) values at the same spatial scales (**Figure 5**). Meanwhile, elevated [CO_2_] also increased the range scale of regular pattern of stomata (**Figure 5**).

### Effects of P supply and [CO_2_] on tissue phosphorus (P), carbon (C), and nitrogen (N) contents of annual ryegrass

Elevated [CO_2_] generally reduced phosphorus contents in both shoots and roots of annual ryegrass (**Table 3**). Specifically, elevated [CO_2_] significantly decreased the phosphorus in shoots by 33.9%, 15.2%, 18.9% and 12.7% under P_0.012_, P_0.06_, P_0.1_ and P_0.5_ (all *p* < 0.05; **Table 3**). However, *e*[CO_2_] only obviously reduced the phosphorus content in roots by 19.9% under P_0.06_ (*p* < 0.05; **Table 3**). In addition, *e*[CO_2_] significantly decreased shoots N by 25.8% and 20.6% under P_0.004_ and P_0.06_ (both *p* < 0.05; **Table 3**), but increased shoots N by 19.1% under P_0.5_ (*p* < 0.05; **Table 3**). Consequently, the C/N ratio in shoot was enhanced by 29.2% and 25.0% under P_0.004_ (*p* < 0.05) and P_0.06_ (*p* < 0.05; **Table 3**) but lowered by 17.9% under P_0.5_ (*p*<0.05; **Table 3**). Additionally, the shoot C (*p* > 0.05), root C (*p* > 0.05), N (*p* > 0.05) and C/N ratio in roots (*p* > 0.05) were barely changed by *e*[CO_2_] regardless of P supply (**Table 3**).

**Table 3:** Effects of elevated [CO_2_] on the tissue phosphorus (P), carbon (C), and nitrogen (N) contents of annual ryegrass under P deficits.

Elements (mg g^-1^)		*a*[CO_2_]											*e*[CO_2_]										
		P_0.004_		P_0.012_		P_0.02_		P_0.06_		P_0.1_			P_0.5_		P_0.004_		P_0.012_		P_0.02_		P_0.06_		P_0.1_		P_0.5_	
**Shoots**	P	0.21 ± 0.02d	0.26 ± 0.03c		0.24 ± 0.02c		0.32 ± 0.02b		0.32 ± 0.02b			0.35 ± 0.01a		0.25 ± 0.01c		0.17 ± 0.01e		0.26 ± 0.02c	0.27 ± 0.01c	0.26 ± 0.02c		0.31 ± 0.02b	
	C	40.9 ± 1.5a	39.4 ± 0.4b		39.0 ± 0.8ab		39.5 ± 1.8ab		39.4 ± 1.2b			40.0 ± 1.3b		39.4 ± 1.0ab		39.4 ± 0.4ab		39.0 ± 0.8b	39.5 ± 1.8ab	39.4 ± 1.2ab		40.0 ± 1.3ab	
	N	3.5 ± 0.3a	2.7 ± 0.2bc		2.7 ± 0.2bc		3.0 ± 0.4a		2.5 ± 0.4bc			2.5 ± 0.2c		2.6 ± 0.2bc		2.8 ± 0.2bc		2.8 ± 0.3bc	2.4 ± 0.3c	2.7 ± 0.2bc		3.0 ± 0.2ab	
	C/N	11.9 ± 0.9d	14.7 ± 1.4abc		14.7 ± 1.6abc		13.5 ± 1.7cd		15.7 ± 2.2abc			16.3 ± 0.9ab		15.4 ± 0.9abc		13.9 ± 0.9cd		14.3 ± 1.7bc	16.9 ± 1.9a	14.8 ± 1.1abc		13.4 ± 0.9cd	
**Roots**	P	3.92 ± 0.24bc	3.91 ± 0.15bc		3.95 ± 0.25bc		4.21 ± 0.37b		4.11 ± 0.22b			4.76 ± 0.18a		3.92 ± 0.09bc		3.88 ± 0.20bc		3.63 ± 0.20cd	3.37 ± 0.34d	3.83 ± 0.18bc		5.10 ± 0.25a	
	C	41.2 ± 0.3a	41.6 ± 0.2a		41.4 ± 0.1a		41.2 ± 0.4a		41.6 ± 0.4a			41.4 ± 0.3a		41.4 ± 0.7a		41.2 ± 0.4a		41.4 ± 0.4a	41.7 ± 0.2a	41.4 ± 0.3a		41.6 ± 0.3a	
	N	1.8 ± 0.1a	1.8 ± 0.1a		1.8 ± 0.1a		1.8 ± 0.1a		1.8 ± 0.1a			1.7 ± 0.1a		1.8 ± 0.1a		1.8 ± 0.1a		1.8 ± 0.1a	1.8 ± 0.1a	1.8 ± 0.1a		1.8 ± 0.1a	
	C/N	23.4 ± 0.3a	22.7 ± 1.1a		23.2 ± 0.6a		22.7 ± 0.8a		22.7 ± 1.1a			23.8 ± 0.8a		22.8 ± 0.9a		23.3 ± 0.3a		23.3 ± 0.6a	22.6 ± 0.5a	23.0 ± 0.3a		22.8 ± 0.6a	

Different lowercase letters indicate significant differences between P deficits treatments at 0.05 level.

The tissue P contents were substantially affected by [CO_2_] (*p* < 0.05) or P supply (*p* < 0.001) from the results of three-way ANOVA (**Table 4**). However, we found statistical differences in the contents of phosphorus (*p* < 0.001), C (*p* < 0.001) and N (*p* < 0.001) as well as the C/N ratio (*p* < 0.001) between tissues (shoot and root) of annual ryegrass (**Table 4**). Moreover, there were obviously interactive effects of [CO_2_] × P supply on the tissue N (*p* < 0.001) and P contents (*p* < 0.05) as well as the C/N ratio (*p* < 0.001; **Table 4**), whereas P supply × tissue only changed the phosphorus content of annual ryegrass (*p* < 0.001; **Table 4**). In addition, our results also showed that [CO_2_] × P supply × tissue significantly changed the contents of N, P and the C/N ratio of annual ryegrass (all *p* < 0.05; **Table 4**).

**Table 4:** ANOVA *p*-values for the effects of P and CO_2_ and interactive effects of P and [CO_2_] on the phosphorus, carbon, and nitrogen contents in tissues of annual ryegrass.

Treatments	Phosphorus	Carbon	Nitrogen	C/N ratio
[CO_2_]	***p*<0.05**	*p* **=**0.549	*p* **=**0.488	*p*=0.678
P supply	***p*<0.001**	*p*=0.447	*p*=0.265	*p*=0.523
Tissue	***p*<0.001**	***p*<0.001**	***p*<0.001**	***p*<0.001**
[CO_2_] × P supply	***p*<0.05**	*p*=0.178	***p*<0.001**	***p*<0.001**
[CO_2_] × tissue	*p*=0.188	*p*=0.759	*p*=0.368	*p*=0.362
P supply × tissue	***p*<0.001**	*p*=0.224	*p*=0.143	*p*=0.115
[CO_2_] × P supply × tissue	***p*<0.05**	*p*=0.067	***p*<0.001**	***p*<0.001**

P<0.05 were considered significant and highlighted in bold.

### Relationships of plant biomass among photosynthesis as well as shoot P and N contents

**Figure 6:**
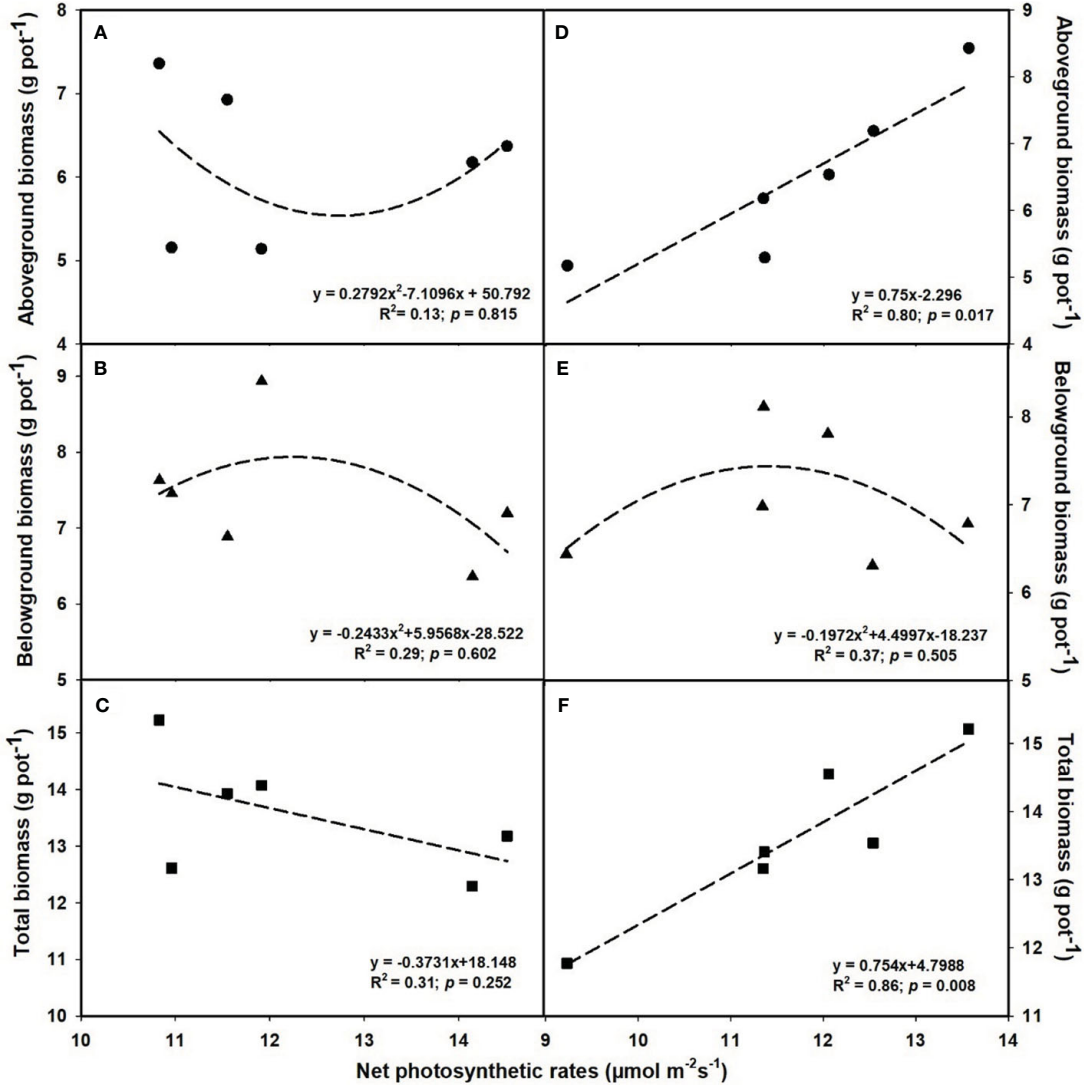
The relationships between net photosynthetic rates and aboveground biomass **(A, D)**, belowground biomass **(B, E)**, and total biomass **(C, F)**. Values are means ± SD (n = 4). The circle symbols represent aboveground biomass, the triangle symbols represent belowground biomass, and the square symbols represent total biomass.

**Figure 7:**
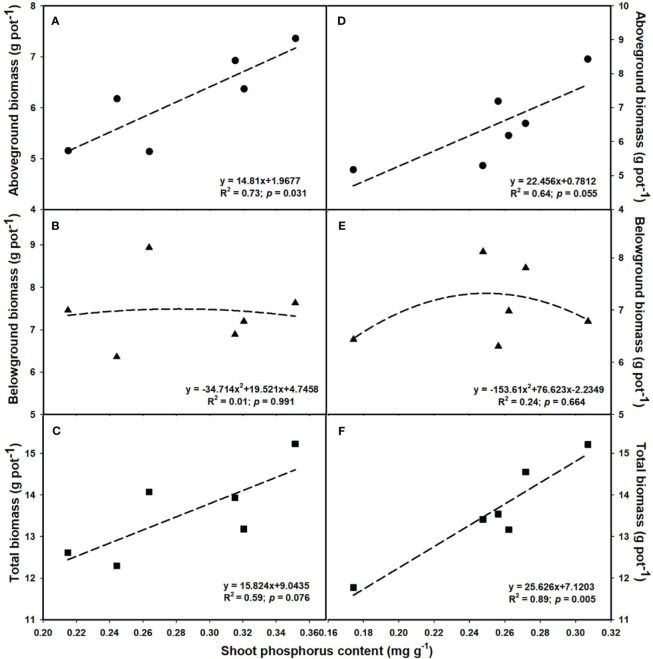
The relationships between leaf phosphorus content and aboveground biomass **(A, D)**, belowground biomass **(B, E)**, and total biomass **(C, F)**. Values are means ± SD (n = 4). The circle symbols represent aboveground biomass, the triangle symbols represent belowground biomass, and the square symbols represent total biomass.

**Figure 8:**
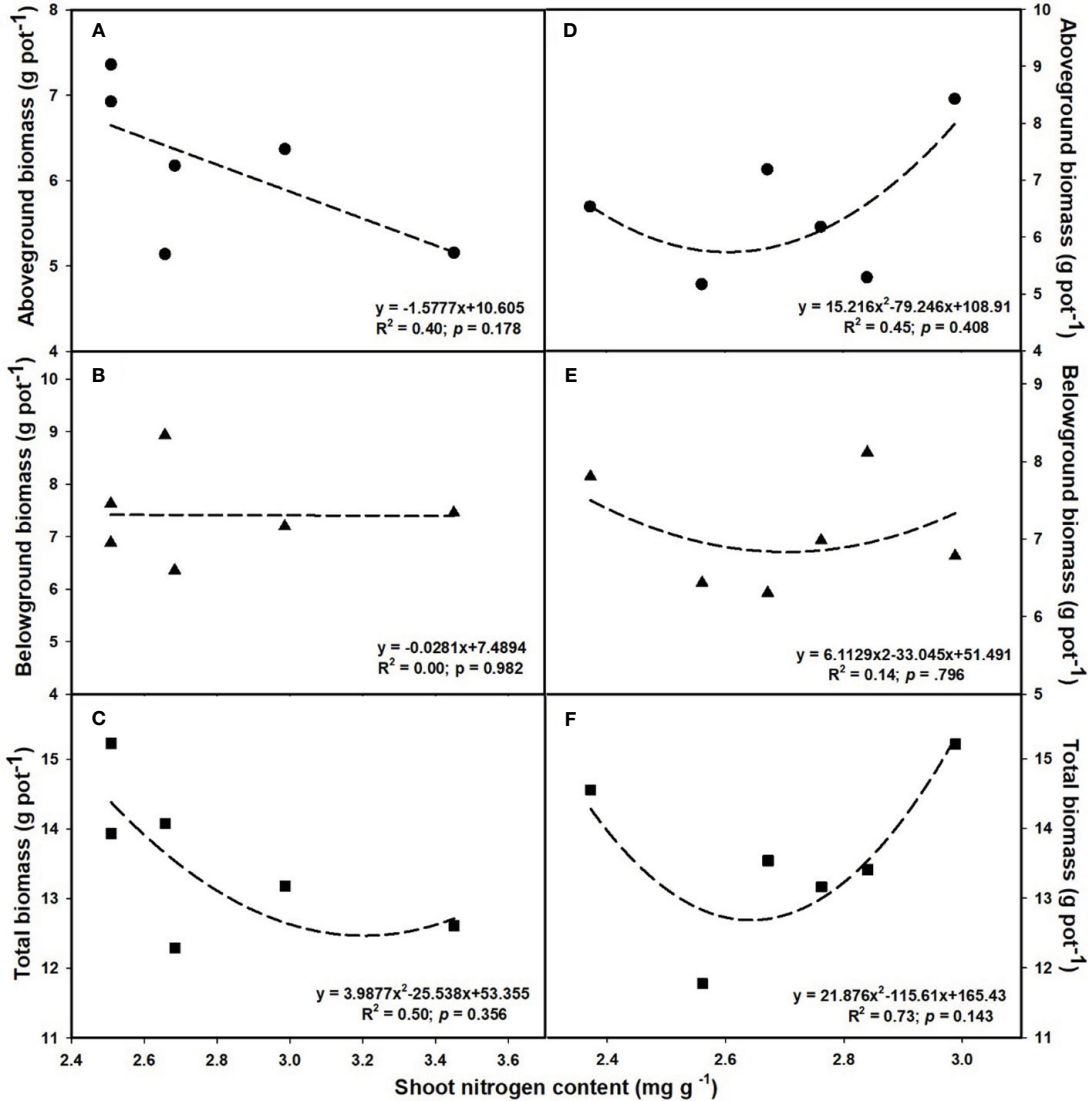
The relationships between leaf nitrogen content and aboveground biomass **(A, D)**, belowground biomass **(B, E)**, and total biomass **(C, F)**. Values are means ± SD (n = 4). The circle symbols represent aboveground biomass, the triangle symbols represent belowground biomass, and the square symbols represent total biomass.

**Figure 9:**
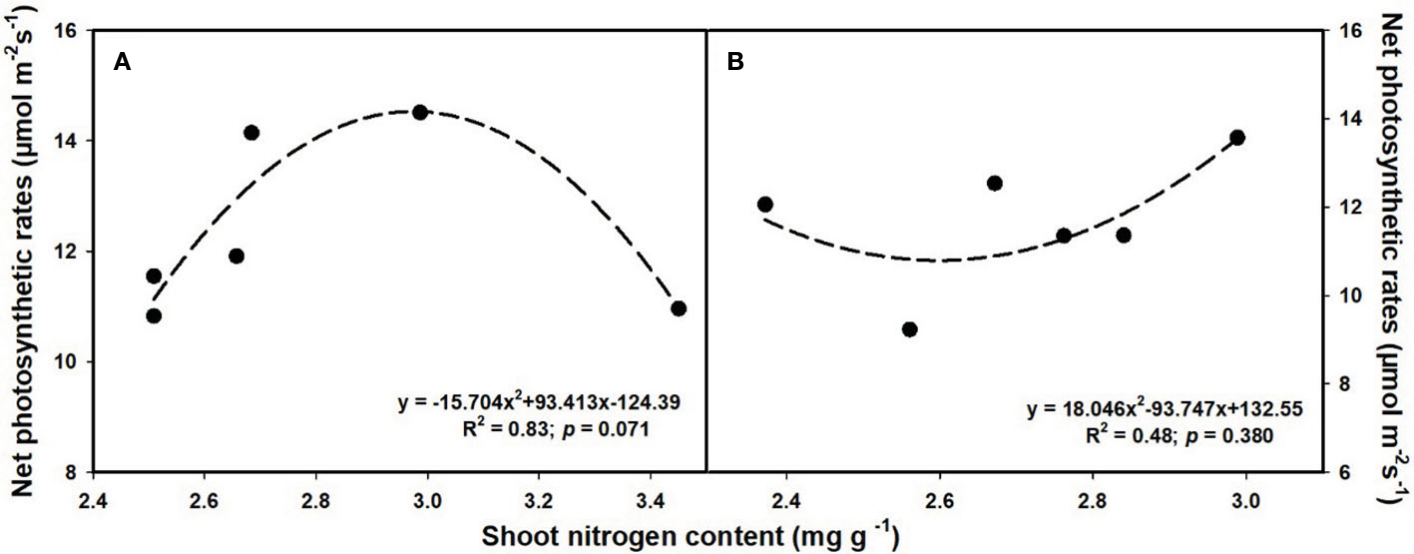
The relationships between leaf nitrogen content and net photosynthetic rates under ambiant **(A)** and elevated [CO^2^] **(B)**.

The aboveground (R^2^ = 0.80, *p* = 0.017; **Figure 6D**) and total biomass (R^2^ = 0.86, *p* = 0.008; **Figure 6F**) demonstrated a linear increase with the elevation of leaf photosynthesis at *e*[CO_2_]. However, no linear or parabolic relationships were found between leaf photosynthesis and aboveground biomass (R^2^ = 0.13, *p* = 0.815; **Figure 6A**), as well as total biomass at *a*[CO_2_] (R^2^ = 0.31, *p* = 0.252; **Figure 6C**). Similarly, we found linear relationships of shoot phosphorus content between aboveground (R^2^ = 0.73, *p* = 0.031; **Figure 7A**) and total biomass (R^2^ = 0.59, *p* = 0.076; **Figure 7C**) at *a*[CO_2_], as well as aboveground (R^2^ = 0.64, *p* = 0.055; **Figure 7D**) and total biomass (R^2^ = 0.89, *p* = 0.005; **Figure 7F**) at *e*[CO_2_]. Nevertheless, there is no obvious correlation between belowground biomass and leaf photosynthesis (**Figure 6**), shoot phosphorus content (**Figure 7**), or shoot nitrogen content (**Figure 8**) regardless of CO_2_ concentration. Moreover, we also found parabolic relationship of shoot nitrogen content with leaf photosynthesis, irrespective of CO_2_ concentration (**Figure 9**).

## Discussion

### P deficit lowers the CO_2_ fertilization effect on the growth of annual ryegrass

Previous studies have established that since the current atmospheric [CO_2_] is suboptimal for the Rubisco enzyme involved in leaf photosynthesis (Ainsworth and Rogers, 2007; Pandey et al., 2015). As a result, the “CO_2_ fertilization effect” would benefit crops, given the enriched atmospheric [CO_2_], leading to plant growth and crop yield (Sakurai et al., 2014; Xu, 2015). For example, a study on winter wheat indicated that increasing [CO_2_] had a maximum boost of more than 50% on its biomass (Butterly et al., 2015). However, it should be noted that most of these studies focused on the effects of *e*[CO_2_] on plant growth and physiological processes were conducted under sufficient nutrition supplies (Li et al., 2007). Therefore, this CO_2_ fertilization effect is likely to be required more essential nutrients for sustaining plant growth (Plénet et al., 2000; Lee et al., 2011; Menge et al., 2012; Pandey et al., 2015; Zheng et al., 2017). Increasing CO_2_ from 400 to 800 μmol mol^-1^ only substantially enhanced the aboveground biomass of annual ryegrass by 14.5% at P_0.5_, indicating that *e*[CO_2_] indeed boosts the plant growth of annual ryegrass with sufficient P supply. However, the aboveground biomass of annual ryegrass subjected to P deficiency was barely affected by elevated CO_2_ and total biomass even obviously decreased by 6.6% at P_0.004_, which suggested that soil P deficiency down regulated the favorable impacts of CO_2_ fertilization effect on annual ryegrass. Moreover, we also found that the belowground biomass was substantially increased under P deficiency, indicating that plants may preferentially distribute more biomass to the roots for nutrient uptake when subjected to P limitation, which is consistent with the conclusions from previous studies that P deficiency may favor root growth more than shoot growth, and thus result in a higher below/above biomass ratio (Péret et al., 2011; Pandey et al., 2015). Additionally, the pronounced interactive effect of CO_2_ concentration and P supply on the aboveground, belowground and the total biomass of annual ryegrass was evident in two-way ANOVA results. The total biomass and aboveground biomass also increased with the increase of P supply, suggesting that the positive CO_2_ fertilization effect on the growth of annual ryegrass was triggered by P supply and the stimulation of biomass accumulation by CO_2_ depends on the P status.

### The CO_2_ fertilization effect on leaf gas exchange under P deficiency

It is well known that CO_2_ is one of the key reactants needed by plants to engage in the biochemical process of photosynthesis, and elevated CO_2_ stimulates leaf photosynthesis (Kimball et al., 2002). However, several lines of evidence suggest that the increased photosynthesis associated with *e*[CO_2_] may be diminished during prolonged exposure, particularly in plants limited by nutrient availability (Lauer et al., 1989; Campbell and Sage, 2006; Reich et al., 2006; Pandey et al., 2015). In this study, *e*[CO_2_] substantially enhanced the net photosynthetic rate at P_0.1_ and P_0.5_ was observed, whereas the net photosynthetic rate of annual ryegrass subjected to P deficit was barely affected or even obviously decreased by elevated CO_2_ concentration, indicating that the stimulating effect of *e*[CO_2_] on the photosynthetic response of plants is weakened as the P concentration decreased. This result may be explained by the fact that high CO_2_ improves uptake efficiency in the presence of adequate P supply (Nilsson et al., 2010; Jin et al., 2011; Pandey et al., 2015). Moreover, Duchein et al. (1993) found in a research on clover (*Trifolium subterraneum* L.) that *e*[CO_2_] obviously increased its net photosynthetic rate under a P concentration supply of 2 mM, while it was inhibited when subjected to P limitation, which was similar to our study. In addition, we found that the highest P supply (0.5 mM) further enhanced the photosynthetic rate under *e*[CO_2_], as evidenced by the higher increase of net photosynthetic rate at P_0.5_ than that of plants treated with P_0.1_, indicating that plants will probably demand ultra-optimal levels of P supply if they want to benefit from the general trend of increasing atmospheric CO_2_ concentration in the future. Moreover, the aboveground biomass of annual ryegrass was barely affected by *e*[CO_2_] when the P supply was lower, although the net photosynthetic rates were higher under P deficit. This suggested that the CO_2_ fertilization effect is more pronounced in stimulating the growth of annual ryegrass under sufficient P supply. Additionally, the obvious decrease in the net photosynthetic rate at elevated CO2, compared to ambient CO_2,_ under the lowest P supply (0.004 mM) likely indicates the crucial role of stomatal limitation on photosynthesis. Furthermore, one of the most consistent responses of plants to elevated atmospheric CO_2_ is a decrease in stomatal conductance (Fleisher et al., 2012; Singh and Reddy, 2014; Zheng et al., 2019; Zheng et al., 2020). This aligned with our results, as we observed a remarkable decline in stomatal conductance with increasing CO_2_ regardless of P supply. However, other nutrient studies have reported that reduced stomatal conductance under lower P supply did not seem to be the major reason for the limitation of photosynthesis (Jin et al., 2011; Singh et al., 2013a). From the above discussion, reduced photosynthesis may be a mechanism by which crops cope with soil phosphorus limitation, which may largely contribute to lower biomass.

### Stomatal diffusion and tissue composition partially explain the decreasing benefit of *e*[CO_2_] to annual ryegrass under P deficiency

It has been reported that plant stomata normally exhibit a variety of short-term behavioral and long-term morphological reactions to CO_2_ (Zheng et al., 2013; Xu, 2015), soil moisture (Sun et al., 2014), thus stomatal regulation is a potential mechanism for adaptation to the external environment during plant growth. In addition to CO_2_ concentrations and soil moisture conditions, Sekiya and Yano (2008) also pointed out that phosphorus supply level can modulate the rate of stomatal production in plant epidermal cells, subsequentially influenced the SD. Our results have further confirmed that stomatal traits of annual ryegrass varied with the P supply and atmospheric CO_2_ concentration. Our results showed that lower P supply reduced the stomatal width and stomatal area, suggesting that annual ryegrass improved their adaptability to different P situations through regulating their stomatal opening. We found that the response of annual ryegrass stomatal density under *e*[CO_2_] depended on P concentration, i.e., stomatal density increased at higher P concentrations and decreased at lower P supply. This CO_2_-induced decrease of stomatal density under lower P supply may explain the downregulation of leaf photosynthesis, since the SD partially determines the efficiency of CO_2_ diffusion from the atmosphere to the mesophyll tissues (Jin et al., 2011). However, observations from previous studies regarding the influence of elevated CO_2_ concentration on SD differed (Ryle and Stanley, 1992; Woodward et al., 2002; Marchi et al., 2004). Regarding this inconsistency, Gray et al. (2000) pointed out that interspecific differences may play a role. However, it should be noted that, as revealed in this study, we cannot deny the strongly interactive effects between environmental variables. Furthermore, it has been well demonstrated that stomatal distribution patterns can affect the net photosynthetic rate and transpiration rate (Soares et al., 2008; Zheng et al., 2013). In the current research, we found that *e*[CO_2_] made the spatial distribution pattern of stomata more regular under sufficient P supply, while the opposite was true when annual ryegrass was subjected to P deficiency. This may partially explain why increasing CO_2_ in this study did not enhance or even lowered the net photosynthetic rate under a lower P supply. This is because the spatial distribution of the stomata on the blade surface affects the diffusion distance of carbon dioxide between the stomata (Zheng et al., 2013; Xu, 2015), which means that the more regular the spatial distribution of the stomata, the more efficient the blade is in terms of gas exchange. Overall, these results implied that P supply partially decided the response of the stomatal distribution pattern to *e*[CO_2_].

It is now well established from a variety of studies that P limitation and *e*[CO_2_] are likely to alter the distribution patterns of tissue constituents as well (Taub and Wang, 2008; Singh et al., 2013a). However, previous studies on leaf P concentration have had contradictory results (Fangmeier et al., 1999; Lewis et al., 2010). For instance, Lewis et al. (2010) reported that leaf P content of *Populus deltoides* was reduced by 22.2-48.6% with increasing the CO_2_ concentration from 350 to 700 μmol mol^-1^. While Fangmeier et al. (1999) found that *e*[CO_2_] did not significantly lower the P concentration in the leaves of wheat. These inconsistencies suggested that the potential complexity of the effects of *e*[CO_2_] on P nutrition. In the current study, we found that the biomass - net photosynthetic rates relationship followed a similar linear or bell-shaped curve like the biomass-shoot P content relationship at the *e*[CO_2_] (**Figures 6** and **7**). It shows that *e*[CO_2_] did not enhance the net photosynthetic rate at a lower P supply, possibly owing to the decrease in leaf P content. Additionally, other studies also suggest that the decrease in nitrogen content in leaves may also be attributed to the decrease in photosynthesis, as the leaf N content is tightly correlated with the content of Rubisco enzymes (Zheng et al., 2019). Moreover, the P content in shoots dramatically decreased under P limitation, but the differences in roots under 0.001 mM - 0.1 mM P supply were mostly insignificant, indicating that shoots may be more sensitive to P limitation than roots.

## Conclusion

We found that the growth enhancement effects of elevated CO_2_ were trivial under the range of P treatments, as amply demonstrated by the reduction in leaf photosynthesis and plant biomass when annual ryegrass was subjected to P limitation. Consequently, P deficiency shifted biomass partitioning by decreasing aboveground production and increasing the root fraction of total biomass. The negative impacts of P limitation on the growth processes of plants benefiting from the effects of CO_2_ fertilization can also be ascribed to the changes in the characteristics of individual stomatal morphology and the stomatal spatial distribution pattern, as well as the changes in tissue composition of annual ryegrass. Nevertheless, the increasing sensitivity of annual ryegrass growth to P supply with increasing [CO_2_] indicates that annual ryegrass will increase its requirements for P to support an aggressive growth response to future atmospheric conditions. Therefore, the role of annual ryegrass in grassland ecosystem responses to future climate change may be incrementally influenced by P supply.

## Data availability statement

The original contributions presented in the study are included in the article/supplementary material. Further inquiries can be directed to the corresponding author.

## Author contributions

FL: Writing – original draft. CH: Writing – original draft. ZC: Formal Analysis, Writing – original draft. CM: Data curation, Writing – original draft. JY: Data curation, Formal Analysis, Writing – review & editing. LL: Formal Analysis, Writing – review & editing. YZ: Data curation, Writing – review & editing. LH: Conceptualization, Writing – review & editing.
